# Gamma activity concentration from building materials: Estimation of gamma absorption and indoor radon concentration in Katsina State, Nigeria

**DOI:** 10.1371/journal.pone.0318497

**Published:** 2025-03-12

**Authors:** Nura Gambo, Ramzun Maizan Ramli, Nurul Zahirah Noor Azman

**Affiliations:** 1 Medical Physics and Radiation Sciences Program, School of Physics, Universiti Sains MalaysiaPenang, Malaysia; 2 Isa Kaita College of Education, Dutsin-Ma, Katsina State, Nigeria; Ural Federal University named after the first President of Russia B N Yeltsin Institute of Physics and Technology: Ural'skij federal'nyj universitet imeni pervogo Prezidenta Rossii B N El'cina Fiziko-tehnologiceskij institut, RUSSIAN FEDERATION

## Abstract

In this research, nineteen (19) samples were collected and analyzed with the following objectives: to evaluate the activity concentration of radionuclides, assess gamma absorption, determine indoor radon concentration, and evaluate the public health impact of building materials used in Katsina State, Nigeria. The study aimed to provide critical data that would inform safe construction practices and regulatory compliance. Samples were sourced locally from various quarry sites, while materials such as cement, paint, tiles, and ceiling materials were purchased from local markets. The methodology involved measuring radionuclide activity concentrations using gamma-ray spectroscopy with a Thallium-doped Sodium Iodide (NaI (Tl)) detector, a highly sensitive method suitable for detecting gamma emissions from radionuclides. Radon gas was identified as the primary radiation source. Results revealed varying activity concentrations of radionuclides across different building materials. Most samples, except for Gravel, Brown Clay (Zone A and C), Kaolin, and Fired Clay Bricks, were below the recommended limits for radionuclide. Similarly, for , except for Cement and Thatch, samples were generally below the average value of 35 Bq/kg. However, several samples including Gravel, Paint, Brown Clay (Zones A, B, C), Thatch, Mud Clay, Laterite, Neem tree, Limestone, Fired Clay Bricks, and Gypsum exceeded the average value of 30 Bq/kg for . The overall average activity concentrations across samples were : 232.421, : 11.791, and : 51.1858 all in Bq/kg. The average Radium equivalent and Gamma index was 113.8 Bq/kg and 0.22, respectively, with an alpha index of 0.11. The external and internal hazard indexes averaged 0.2292 and 0.3102, indicating that these materials pose no significant radiological health risk when used in construction, as all values are below international guidelines of 370 Bq/kg and 1 mSv/y. This study concludes with a recommendation for public awareness on the effects of radiation and the need for continued monitoring and regulation of radiation exposure. The significance of this study lies in its contribution to public health and safety, supporting regulatory compliance and helping to prevent potential health risks associated with construction materials.

## Introduction

Public exposure to natural radioactivity, primarily from radon gas and gamma radiation, poses significant health risks, particularly through construction materials derived from natural raw resources. These materials may contribute to increased indoor radiation absorption rates, leading to potential health hazards from both internal and direct gamma exposure [1,2]. While extensive studies have been conducted globally, including in regions like Saudi Arabia where a dose rate of approximately 59 nano grays per hour has been observed in building materials [3], such detailed data are lacking for Katsina State, Nigeria. This gap highlights the need for region-specific research, as the concentration of Natural Occurring Radioactive Materials (NORMs) such as uranium-238, thorium-232, and potassium-40 can vary significantly based on local geology and materials used in construction [4,5].

Previous research has established the importance of evaluating gamma radiation levels in building materials, as prolonged exposure can lead to serious health conditions, including tissue and cellular damage [6]. Research carried out in different areas has confirmed that typical building materials such as granite, concrete, bricks, and tiles contain gamma-emitting radionuclides [5], however, there is a noteworthy lack of information on Katsina State. In order to fill this gap, this study analyzes the activity concentrations of these radionuclides in locally produced construction materials from this area. By doing so, it can determine whether or not these materials comply with safety regulations and whether or not there are any possible threats to the public’s radiological health.

Radon gas, a byproduct of uranium-238 decay, has been found to be a significant cause of dangers to public health and indoor air quality, especially in buildings with inadequate ventilation [6]. Previous studies have shown that radon absorption significantly contributes to the ionizing radiation dose received by individuals, and has been linked to increased respiratory health risks and mortality rates [7,8,9]. But it’s still unknown how much radon is present in Katsina State due to construction materials. In order to close this knowledge gap, this study will explicitly evaluate the levels of radon in building materials that are often utilized in this area. This will provide important information that might guide local building practices and public health regulations. Previous studies have linked greater mortality and respiratory health risks to radon absorption, which significantly raises a person’s exposure to ionizing radiation [10]. The degree of Katsina State’s radon exposure from construction materials is yet unknown, though. This study aims to fill this knowledge vacuum by accurately assessing the radon levels in commonly used construction materials in this region. The data provided might have a significant impact on local building standards and public health campaigns. The literature on the topic highlights how crucial it is to monitor and regulate naturally occurring radioactivity in building materials in order to protect the general public’s health. Building on earlier findings, this study provides a focused assessment of the radiological implications for interior areas in Katsina State. bringing a regional perspective to the global understanding of natural radioactivity and its impact on human health.

## Natural radioactivity in building materials

Common construction materials like concrete, bricks, tiles, and granite often incorporated with uranium-238, thorium-232, and potassium-40. These radionuclides emit gamma radiation absorbed by people inside buildings [11].

## Gamma radiation and health implications

Gamma radiation from natural radionuclides is significant source of radiological hazard. This poses substantial damage to public, particularly in indoor environments where people spend considerable time. Prolonged exposure to elevated gamma radiation levels can damage living tissues and cells, leading to serious health conditions. Therefore, evaluating gamma activity concentration in building materials is crucial to ensure compliance with safety standards [12].

The decay product of uranium-238 is dangerous that can accumulate in indoor environments, especially in poorly ventilated buildings. Radan gas constitutes a significant portion of the ionizing radiation dose received by individuals [13].

Previous studies have investigated the exposure to radionuclides in building materials and in Saudi Arabia, results indicated the presence of approximately 59 nano grays per hour of dose rate [14]. Other studies also found the relationship between internal exposure and health hazards and indicated that radon absorption accounts for a significant number of deaths [15].

## Radon gas and indoor air quality

Radon gas exists in the air in large quantities and the inhalation rate is increases. People living in dwellings are vulnerable to ingestion of higher doses. Large quantities of radon are found in radium atoms. This radon gas seeps into the troposphere, and when inhaled it becomes hazardous to human health [5].

### Problem statement

Building materials in Katsina State may contain naturally occurring radionuclides (NORM) such as potassium-40, thorium-232 and radium-226. The radiation emitted by these materials can pose health risks to the inhabitants and there is inadequate information on the effect of NORM from building materials*.*

#### Aims of this research.

The research was aimed to:

- evaluate the activity concentration of radionuclides
- assess gamma absorption
- determine Indoor Radon Concentration
- evaluate Public Health Impact

#### Research questions

- What are the activity concentrations of natural occurring radioactive materials (NORMs) such as uranium-238, thorium-232, and potassium-40 in building materials commonly used in Katsina State, Nigeria?
- How do the measured gamma radiation levels in these building materials compare to international safety standards for radiological health?
- What is the concentration of indoor radon gas associated with the use of these building materials, and how does it contribute to overall radiation exposure in residential buildings in Katsina State?
- What are the potential health risks, particularly related to gamma radiation and radon exposure, for occupants of buildings constructed with these materials in Katsina State?

### Background of the study

Because it covers a number of significant topics regarding the radiological safety of the building materials used in Katsina State, the literature evaluation is crucial to our investigation.

Understanding the concentrations of naturally occurring radioactive materials (NORMs) such uranium-238, thorium-232, and potassium-40 is crucial. Reading through the literature that has already been written on these materials may help us understand more about the radiation levels in commonly used building materials in Katsina State. To evaluate the potential radiation dangers that these materials may pose, this knowledge is crucial. Comparing the observed γ radiation doses from these building materials to international safety standards is also essential through comparative analysis, we can determine whether the building’s materials meet international safety requirements and ensure that residents aren’t subjected to unnecessary health risks. Furthermore, this comparison provides a basis for safety protocols and legislation, which might facilitate the creation of local policies and regulations. The amount of radon gas present indoors is a significant additional problem. Analyzing radon levels and their contribution to overall radiation consumption in residential buildings is crucial for identifying potential health risks. Knowing the levels of radon aids in selecting the most effective mitigation techniques to protect occupants from the harmful effects of indoor radiation. One important factor in indoor radiation exposure is radon. Finally, reviewing the literature makes it easier to pinpoint potential health risks related to radon and gamma radiation exposure. To assess the long-term impact on renters’ health, it is critical to understand these risks. Health recommendations and regulations for safe building practices must be created in order to lessen the risks associated with radiation exposure and ensure the residents of Katsina State are safe. This content supports such initiatives [16].

### Literature review

Gebeyehu et al. (2021) examined the concentration of natural radioactivity in iron ore deposits in Ethiopia. Their findings suggested that the materials were suitable for construction, with radiation hazards remaining below international safety limits. Similarly [17].

Islami Rad et al. (2023) assessed soils used in construction in Qom, Iran, identifying variations in thorium-232, radium-226, and potassium-40 levels, which highlight the importance of localized studies in radiation safety [18].

Jasaitis and Pečiulienė (2021) focused on radon exhalation from building materials in Lithuania, particularly in underground parking lots. Their work established a strong correlation between radium activity and radon concentrations, emphasizing the need for monitoring in confined spaces [19].

Legasu et al. (2022) extended these findings by evaluating radiological risks from building materials in Dessie City, Ethiopia, underscoring regional disparities in exposure risks [20]

Leagsu et al. (2021) conducted a detailed study on the natural radioactivity levels and associated health risks in Delanta-Dawunt, Wollo District. This region is known for its unique geological formations, which are suspected to harbor elevated concentrations of radionuclides. The study identified significant levels of potassium-40 (40K), radium-226 (226Ra), and thorium-232 (232Th),with absorbed dose rates that exceeded the global average limits recommended by UNSCEAR (2000) [21].

Sidi et al. (2023) evaluated radiation protection measures in Kano, Nigeria, emphasizing the need for adherence to safety protocols in medical and construction settings. Sopoh et al. (2022) explored compliance with safety protocols in Benin’s medical imaging units, further demonstrating the interdisciplinary relevance of radiation studies [22].

Sopoh et al. (2022) conducted a detailed study assessing compliance with radiation safety measures in medical imaging units in Southern Benin. The findings highlighted a need for improved safety culture, periodic training, and updated safety protocols to meet international standards [10].

Kannan et al. (2019) conducted a comprehensive investigation into alpha decay rates in heavy elements, contributing significantly to the theoretical understanding of radionuclide behavior. Their work emphasized the quantum mechanical nature of alpha decay, providing a detailed analysis of tunneling probabilities and decay lifetimes for isotopes with high atomic masses, such as uranium, thorium, and radium [23].

Lu et al. (2022) conducted a comprehensive study in Canada, focusing on external exposure levels and identifying critical thresholds to ensure environmental safety. Their research provided valuable insights into radiation levels from soil, rocks, and building materials, emphasizing the need for regular monitoring to mitigate potential health risks [24]

Islam et al. (2021) emphasize the implementation of IAEA standards to establish robust nuclear safety infrastructure. Their study outlines the challenges in meeting safety protocols and developing local expertise for regulatory and operational roles [25].

Oladotun et al. (2022) evaluated the radiological health risks associated with Indian tiles available in Nigerian markets. The researchers measured the activity concentrations of naturally occurring radionuclides-namely , , and in seven imported Indian tile samples. The mean activity concentrations were found to be 68.03 Bq/kg for 84.79 Bq/kg for h, and 620.89 Bq/kg for . These values exceed the recommended safety limits for building materials. Despite the elevated activity concentrations, other radiological parameters, such as radium equivalent activity, hazard indices, and annual effective dose, were within safe limits, suggesting that the tiles are generally safe for construction purposes in Nigeria. The study recommends regular monitoring to ensure consumer safety, given the reliance on imported materials [26].

The study by Sani (2023) evaluated the elemental composition of kaolin from Katsina State, Nigeria, highlighting its significance for radiation safety and industrial applications. This research has been foundational in understanding the mineralogical properties and environmental implications of using kaolin, particularly in the context of radiation shielding and safety [27].

Olowookere et al. (2022) conducted a comprehensive evaluation of absorbed radiation doses in various Nigerian institutions, proposing regulatory measures for enhancing long-term safety. This review highlights key findings and related research, offering a broader perspective on radiological safety and policy implications [28].

Tien et al. (2022) focuses on modeling radioactive decay in molten salts, aiming to enhance radiation detection methods in non-traditional environments. Molten salts are pivotal in advanced nuclear reactors and reprocessing technologies due to their superior thermal properties and capacity to dissolve various nuclear materials. Understanding radioactive decay within these media is essential for effective monitoring and safety [29].

The study by Omeje et al. (2024) represents a significant step in integrating ecological considerations into radiological research. It emphasizes the need for multidisciplinary approaches to address marine sediment contamination, balancing ecological preservation with public health protection [30].

The study by Ademola and Abdulkareem (2019) provides valuable insights into radon levels in Lagos State’s groundwater, indicating that the health risks associated with radon ingestion are minimal in the surveyed areas. However, continuous monitoring and public awareness are essential to ensure water safety, considering the variability of radon concentrations influenced by geological factors [31].

Noska et al. (2022) discuss the variation in radiological safety practices across regions. Their framework for harmonizing international standards underscores the importance of integrating local practices with globally accepted safety norms to ensure equitable radiation protection [32].

The comparative study by Abraham et al. (2019) provided a significant framework for understanding and harmonizing radiological safety standards globally. Their work has inspired subsequent research and discussions on mitigating radiological risks while ensuring adherence to standardized safety protocols. This literature review synthesizes key findings from related works to expand on the implications and applications of their research [33].

Garba et al. (2023) conducted a critical evaluation of local building materials in Northwestern Nigeria, emphasizing radiological safety and advocating for stricter regulations. The study highlights the radiological risks associated with primordial radionuclides in common materials such as sand, clay, kaolin, and gypsum [34].

Al-Mur et al. (2022) investigated marine sediments along the Saudi Arabian coast, focusing on their chemical composition, ecological risks, and potential contamination sources. Their work has been pivotal in understanding the impacts of natural and anthropogenic activities on the Red Sea’s marine ecosystems. This literature review synthesizes key findings from their research and related studies [35].

Hazou & Patchali (2021) used multivariate statistical tools to analyze radionuclide dispersion and assess risks associated with phosphate mining near Kpémé. Their findings informed local policymakers about implementing risk mitigation strategies [36].

Orabi et al. (2019) presented a pivotal review of indoor radiation modeling techniques, addressing the critical need for predictive tools in sustainable and safe building designs. The study emphasized integrating computational tools to predict and mitigate radiation exposure risks within indoor environments [37].

Verde et al. (2021) contribute valuable insights into the prevalence of indoor radon concentrations in Southern Italy, highlighting the variability of radon levels across different dwellings and the associated health risks. The study emphasizes the importance of continuous monitoring and mitigation strategies to minimize radon exposure in residential areas, particularly in regions with high radon levels. By linking radon concentration data with effective doses and cancer risk estimations, the study serves as an important resource for public health and environmental safety in the context of radon exposure [38]

Abdel Gawad et al. (2024) make significant contributions to the field of radon exhalation measurement by presenting innovative methods for assessing radon release from various sources. Their study enhances the accuracy and reliability of radon exhalation rate measurements, which are essential for understanding exposure risks and mitigating the public health threat posed by radon. By combining direct and indirect measurement techniques, improving calibration procedures, and integrating these methods into broader risk assessment models, their work provides a foundation for more effective radon monitoring and mitigation strategies worldwide [39].

Mphaga et al. (2024) provide valuable insights into the risks posed by radon exposure near gold mine tailings in South Africa. Their study underscores the significance of site-specific interventions to mitigate radon emissions and protect local populations from the associated health risks. By developing tailored mitigation strategies, improving monitoring practices, and fostering greater awareness about radon exposure, their research contributes to the ongoing efforts to reduce the public health impact of radon in mining communities. Their work highlights the need for comprehensive, regionally adapted approaches to managing radon risks and protecting vulnerable populations from the long-term health consequences of exposure [40]

## Materials and methods

In this research, the area of study was discussed, mathematical model was employed to assess the radiological safety and determine the rate of radiation dose obtained from buildings. This approach aimed to ensure the safety in the building materials and maintenance.

### Study area

Katsina State is in northeastern Nigeria, positioned between latitudes 11°07’N and 13°22’N and longitudes 6°52’E and 9°20’E. It shares a border with the Niger Republic to the north, Kaduna State to the south, Kano and Jigawa States to the east, and Zamfara State to the west. The state comprises 34 Local Government Areas and has a population of approximately 5,801,584 people. Katsina State spans an area of 24,192 square kilometers (9,341 square miles).

Geologically, Katsina State is part of the extensive sedimentary formations of the Chad Basin and the Sokoto Basin. The state’s geological formations include Precambrian basement complex rocks, which are overlain by sedimentary deposits from the Cretaceous to the Quaternary periods. These formations are rich in a variety of mineral resources, including gold, kaolin, asbestos, silica sand, marble, quartz, talc, precious stones, granite, and feldspar. The sedimentary rocks in the region consist mainly of sandstone, shale, and clay, which play a significant role in the state’s mineral wealth.

The state’s topography is predominantly flat to gently undulating, with occasional isolated hills and inselbergs. The presence of these geological features and mineral deposits makes Katsina a region of interest for both geological studies and economic exploration (2006 census).

### Sample collection and sample preparation

#### Sample details.

In this study, we analyzed 19 samples representing various building materials commonly used in Katsina State, Nigeria. These samples were sourced from distinct locations, including quarry sites and local markets, and are categorized as follows:

1. Samples from Quarry Sites:

Gravel, Brown Clay (Zones A, B, C), Kaolin Clay, Thatch, Mud Clay, Laterite, Gypsum, Sand, Fired Clay Bricks.

1. Samples from Local Markets

Cement (Dangote), Tile (Time Ceramic), Paint (Fine Clear Paint) and Ceiling Materials (Nigerian made)

1. Organic Materials from Local Sources

Neem Tree, Baobab Tree, and Cow Dung

#### Sampling locations and strategy.

Building material samples were collected from various zones across Katsina State, ensuring geographic and material diversity. The study focused on three primary zones, encompassing urban, semi-urban, and rural. Each zone included multiple sampling sites to capture the full spectrum of local construction practices:

**Zone A (Central)**: Dutsin-Ma, Katsina, Batsari, Danmusa, Batagarawa, and Safana.
**Zone B (North)**: Daura, Baure, Sandamu, Zango, and Mai’adua.
**Zone C (South)**: Funtua, Malumfashi, Kankara, Faskari, Musawa, and Dandume.

The selection of these zones was carefully designed to encompass both modern and traditional construction techniques prevalent across the state. Additionally, key markets were included to provide commercial building materials that accurately represent local commerce and current construction trends [40].

**Central Market, Katsina City**: Supplied cement, paint, and ceiling materials.
**Dutsin-Ma Market**: Provided tiles.
**Funtua Market**: Supplied kaolin, commonly used in semi-urban settings.
**Malumfashi Market**: Offered kaolin and paint for finishing work.
**Daura Market**: Provided cement and other widely-used materials

#### Description of sampled materials.

The sampled materials were selected for their relevance to gamma activity measurements and their roles in local construction. These include a combination of industrial products and traditional materials, categorized as follows:

#### Modern industrial materials

**Cement (Dangote Cement)**: Widely used in foundations, walls, and structural elements.
**Tiles (Time Ceramic)**: Utilized for flooring and wall finishes.
**Ceiling Materials**: Includes imported varieties for residential and commercial use.
**Paint (Fine Clear Paint)**: Commercial paints for interior and exterior finishes.
**Gypsum**: Applied in ceiling boards and wall plaster, noted for gamma absorption.
**Sand**: A key component in concrete and mortar mixtures.
**Gravel**: Essential for concrete and foundational work.

#### Traditional materials

**Brown Clay and Mud Clay**: Sourced from all zones, and used in traditional construction.
**Kaolin**: A fine clay known for its flexibility and durability.
**Thatch**: Organic roofing material evaluated for gamma absorption.
**Laterite**: Reddish soil valued for its strength in construction.
**Neem Tree Wood and Baobab Tree Wood**: Organic materials for structural and decorative use.
**Cow Dung**: Mixed with clay in rural construction.
**Limestone**: Key for cement production and gamma activity measurement.
**Fired Clay Bricks**: Common in wall construction.

#### Market names and locations of purchased items.

To complement the site-collected materials, additional samples were obtained from key markets across Katsina State:

Central Market, Katsina City: Provided a selection of cement, paint, and ceiling materials, essential components for various construction projects.
Dutsin-Ma Market: Offered locally sourced tiles.
Funtua Market: Supplied kaolin, a material commonly used in semi-urban construction projects.
Malumfashi Market: Provided kaolin and paint, frequently used in local finishing applications.
Daura Market: Supplied a variety of construction materials, including cement suitable for diverse building types.

This comprehensive selection facilitates a thorough assessment of gamma activity and radon potential across various building materials. The information enhances the methodology section by detailing the sources and geographic distribution of the sampled materials, providing clarity and strengthening the study’s foundation.

#### Sample characteristics.

When collecting samples, these characteristics were considered, texture, finish, color variations, and packaging. For instance, cement should have a fine, lump-free texture. Tiles should have a uniform glaze for a consistent appearance. Ceilings were made from high-quality materials that provide both aesthetic appeal and sound insulation, and they may come in various designs, including decorative and plain styles. A paint sample has the formation of coverage and drying time, with a clear finish that offers protection while enhancing the surface. Market characteristics such as Dutsin-Ma Central Market, and Katsina Central Market known for a variety of construction materials, providing both local and imported goods serving the local community and surrounding areas.

These samples underwent a process of crushing into fine powder using a mechanical crusher, followed by sieving to achieve particle sizes consistent with a 500 μm mesh for uniformity. Approximately 300 g of each sample was meticulously transferred into 350 ml beakers and sealed. The inner surfaces of these containers were treated with a layer of Vaseline and candle wax to prevent any potential contamination and seal tape was employed to secure the container caps Subsequently, the samples were meticulously weighed between 250-300 g, and sealed in containers designed to match the geometry of the NaI (TI) detector, dried at a temperature of 30℃ with a mean relative humidity of 70%. The sealed containers were then left for 90 days to ensure the attainment equilibrium for uranium series of radium-226, radon-222 and potassium-40.

This research did not require official permission for sample collection since samples were locally sourced from quarry sites where permission is not mandatory. Additionally, seven samples cement, tile, ceiling material, gravel, paint, kaolin, and limestone were obtained from local markets, where no official permission was needed.

#### Experimental analysis.

The samples were analyzed using gamma-ray spectrometry with a sodium iodide (NaI (Tl)) detector. The measurements were conducted in the Center for Energy Research and Training (CERT) at Ahmadu Bello University, Zaria, Nigeria. The spectrometer setup included a shielded Canberra NaI(Tl) sensor housed within a 100 mm thick lead block, designed to minimize background radiation. The detector, manufactured by Canberra Inc., USA (Model 802-series, Model No. 727 SN: 11914167), offers a resolution of approximately 80 percent at 662 keV from cesium-137 and at 1173 keV and 1332 keV from cobalt-60. The gamma-ray energies utilized for measurements were 1460 keV and 2614 keV, corresponding to potassium-40 and thallium-208 in the thorium series, respectively. The detector dimensions were 7.62 cm x 7.62 cm, housed in a 6 cm thick lead shield lined with cadmium and copper panes.

Before analyzing the samples, a detailed calibration of the gamma-ray spectrometer was performed. The calibration involved using standard gamma-ray sources such as cesium-137 with an energy of 662 keV, cobalt-60 with energies of 1173 keV and 1332 keV, and potassium-40 with an energy of 1460 keV. These sources were used to establish an energy-channel relationship by mapping the known gamma-ray energies to the corresponding channels on the multichannel analyzer (MCA). The detector’s efficiency was calibrated by calculating the ratio of the observed counting rate to the expected number of gamma rays at different energy levels, creating an efficiency curve that was used to correct the measured spectra for variations in detector efficiency across the energy spectrum.

The calibration process also included measuring background radiation, which was subtracted from the spectra obtained during actual sample analysis to ensure accurate results. Reproducibility was confirmed through periodic quality checks, and the calibration was validated using additional check sources.

The energy nuclides (radionuclides) involved in this analysis are those that emit gamma rays detectable by the NaI (Tl) detector at the specified energy levels. The radionuclides include cesium-137 at 662 keV, cobalt-60 at 1173 keV and 1332 keV, potassium-40 at 1460 keV, radium-226 at 186 keV, and thorium-232 with notable peaks, including 2614 keV from thallium-208. The spectra displayed gamma-ray energy peaks, which were compared with known values to identify the specific radionuclides present. The multichannel analyzer was utilized to convert the analog signals from the detector into digital signals, which were then processed and displayed as spectra, allowing for the accurate identification and quantification of the radionuclides present in the samples [40].

#### Parameters evaluated.

The study analyzed the following:

- Activity concentrations of potassium-40, thorium-232, and radium-226.
- Gamma radiation absorption rates and radon gas emissions.
- Radiological hazard indices (external, internal, gamma, and alpha indices).
- Annual effective dose rates for indoor and outdoor environments.

### NORMS–related radiological hazard in the samples

#### Gamma indices.

The gamma index was used to calculate the amount of gamma absorption due to direct gamma exposure to radiation, and represented by the Eq (1)


$$I_{\gamma }=\frac{C_{Ra}}{300Bq/Kg}+\frac{C_{Th}}{200Bq/Kg}+\frac{C_{K}}{3000Bq/Kg}$$ (1)

Where,

$I_{\gamma }$ is the Gamma Index

$C_{Ra}$ is Radium concentration

$C_{Th}$ is Thorium concentration

$C_{K}$ is Kalium concentration

#### Alpha indices.

The amount of radon gas inhaled or internally exposed is determined using the alpha index. Eq (2) was applied in this study and used to determine the radon absorption.

Alpha Index (I_*α*_):


$${\text{I}}_{\alpha }=\frac{C_{Ra}}{200Bq/Kg}$$ (2)

External hazard index ($H_{ex})$:


$$H_{ex}=\frac{C_{Ra}}{370}+\frac{C_{Th}}{259}+\frac{C_{K}}{4810}\leq 1$$ (3)

While Internal hazard index ($H_{in}$):


$$H_{in}=\frac{C_{Ra}}{185}+\frac{C_{Th}}{259}+\frac{C_{K}}{4810}\leq 1$$ (4)

#### Estimation of absorbed rate.

In this research, the absorbed dose received by the populace due to gamma emissions from $232_{\text{Th}}$, $226_{\text{Ra}}$, and $40_{\text{K}}$Bq/Kg is being investigated. Eq (5) was used. Eq (6) represented the indoor absorbed dose. Eq (7) represented the outdoor absorption in (nSv). Eq (8) was defined the Radium equivalent (${\text{Ra}}_{\text{eq}})$ in Bq/Kg [13].

Absorbed dose (D):


$$D\left(nGyh^{-1}\right)=0.462C_{Ra}+0.604C_{Th}+0.0417C_{K}$$ (5)

$$nGy^{-1}\times 8760h\times 0.8\times 0.7SvGy^{-1}$$ (6)

$$nGy^{-1}\times 8760h\times 0.2\times 0.7SvGy^{-1}$$ (7)

Radium equivalent $\left({\text{Ra}}_{\text{eq}}\right)$:


$${\text{Ra}}_{\text{eq}}=C_{Ra}+1.43\;{\text{C}}_{\text{Th}}+0.077{\text{C}}_{\text{K}}$$ (8)

## Result and discussion

The quantification of radionuclides from building materials were measured presented in Table 1. The gamma absorptions were recorded in Table 2. However, the findings from this study highlight the variability in radionuclide concentrations in building materials commonly used in Katsina State.

**Table 1:** Activity concentration in building materials in Bq/Kq.

S/N	Samples	Sample Identification	Concentration of K-40		Concentration of Ra-226		Concentration of Th-232	
			values	Error ±	values	Error ±	values	Error ±
1	Cement	A	230.94	7.51	37.88	1.15	21.78	0.68
2	Tile	B	209.48	9.82	23.75	2.20	14.82	2.28
3	Ceiling	C	172.16	5.44	18.99	3.47	18.13	1.36
4	Gravels	D	482.12	9.64	23.63	3.75	75.48	3.42
5	Paint	E	270.92	6.84	19.92	2.43	155.87	9.23
6	Brown Clay -Zone A	F	489.74	6.06	22.13	1.50	56.44	5.24
7	Brown Clay -Zone B	G	130.79	6.22	18.77	1.39	81.64	4.21
8	Brown Clay -Zone C	H	360.81	6.99	20.97	1.73	53.71	5.13
9	Kaolin	I	455.21	7.68	32.44	3.01	29.76	1.71
10	Thatch	J	104.90	3.80	41.22	5.10	65.21	2.61
11	Mud clay	K	293.10	7.61	15.33	0.89	40.34	1.58
12	Laterite	L	233.21	4.31	11.41	3.80	52.68	1.81
13	Neem tree	M	71.67	1.80	13.49	1.28	44.71	1.90
14	Limestone	N	201.90	7.55	19.61	5.90	44.22	2.11
15	Cow dung	O	98.51	3.61	30.21	3.45	2.81	2.00
16	Gypsum	P	80.57	5.57	18.24	5.22	62.80	1.27
17	Sand	Q	71.62	7.24	10.22	0.83	40.66	1.61
18	Fired clay Bricks	R	316.16	7.55	35.44	1.99	80.89	1.90
19	Baobab tree	S	144.72	5.12	18.33	6.97	30.89	1.61

**Table 2:** External and internal hazard index, gamma and alpha index of the radionuclides of the samples.

S/N	Samples	External hazard index $\left(H_{ex}\right)$	Internal hazard index $\left(H_{in}\right)$	Gamma index $\left(I_{\gamma }\right)$	Alpha index $\left(I_{\alpha }\right)$
1	Cement	0.2345 ± 0.0248	0.3368 ± 0.0451	0.3122	0.1894
2	Tile	0.1649 ± 0.0355	0.2291 ± 0.0621	0.2231	0.1188
3	Ceiling	0.1571 ± 0.0284	0.2085 ± 0.0431	0.2114	0.0950
4	Gravel	0.4555 ± 0.0413	0.5193 ± 0.0673	0.6169	0.1182
5	Paint	0.7112 ± 0.0298	0.7650 ± 0.0483	0.9361	0.0996
6	Brown Clay -Zone A	0.3796 ± 0.0233	0.4393 ± 0.0397	0.5192	0.1107
7	Brown Clay -Zone B	0.3932 ± 0.0231	0.4440 ± 0.0399	0.5144	0.0939
8	Brown Clay -Zone C	0.3391 ± 0.0267	0.3958 ± 0.0456	0.4588	0.1049
9	Kaolin	0.2972 ± 0.0328	0.3849 ± 0.0535	0.4086	0.1622
10	Thatch	0.3878 ± 0.0333	0.0698 ± 0.0438	0.1438	0.2121
11	Mud clay	0.2575 ± 0.0245	0.0847 ± 0.0451	0.1301	0.0771
12	Laterite	0.2831 ± 0.0275	0.0749 ± 0.0382	0.1130	0.0568
13	Neem tree	0.2831 ± 0.0082	0.0395 ± 0.0145	0.0689	0.0669
14	Limestone	0.2654 ± 0.0470	0.0694 ± 0.0637	0.1152	0.0979
15	Cow dung	0.1128 ± 0.0818	0.0378 ± 0.1463	0.0851	0.1512
16	Gypsum	0.3086 ± 0.0370	0.0511 ± 0.0548	0.0907	0.0916
17	Sand	0.1989 ± 0.0248	0.0360 ± 0.0479	0.0603	0.0510
18	Fired clay Bricks	0.4746 ± 0.0316	0.1165 ± 0.0542	0.1966	0.1773
19	Baobab tree	0.1985 ± 0.0412	0.0515 ± 0.0548	0.0899	0.0916

**Fig 1:**
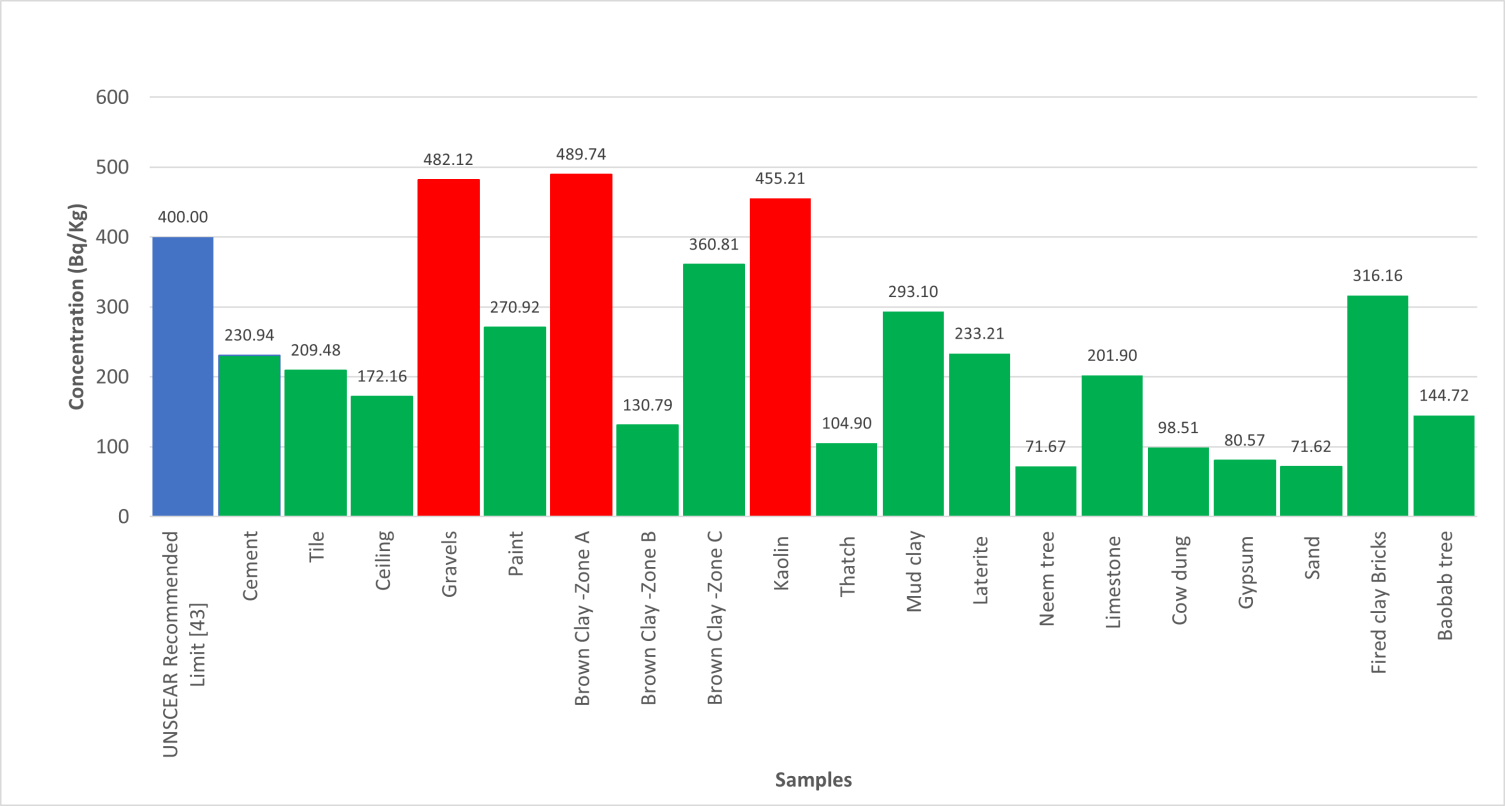
Illustrate the activity concentrations of $40_{\text{K}}$ across the analysed samples.

**Fig 2:**
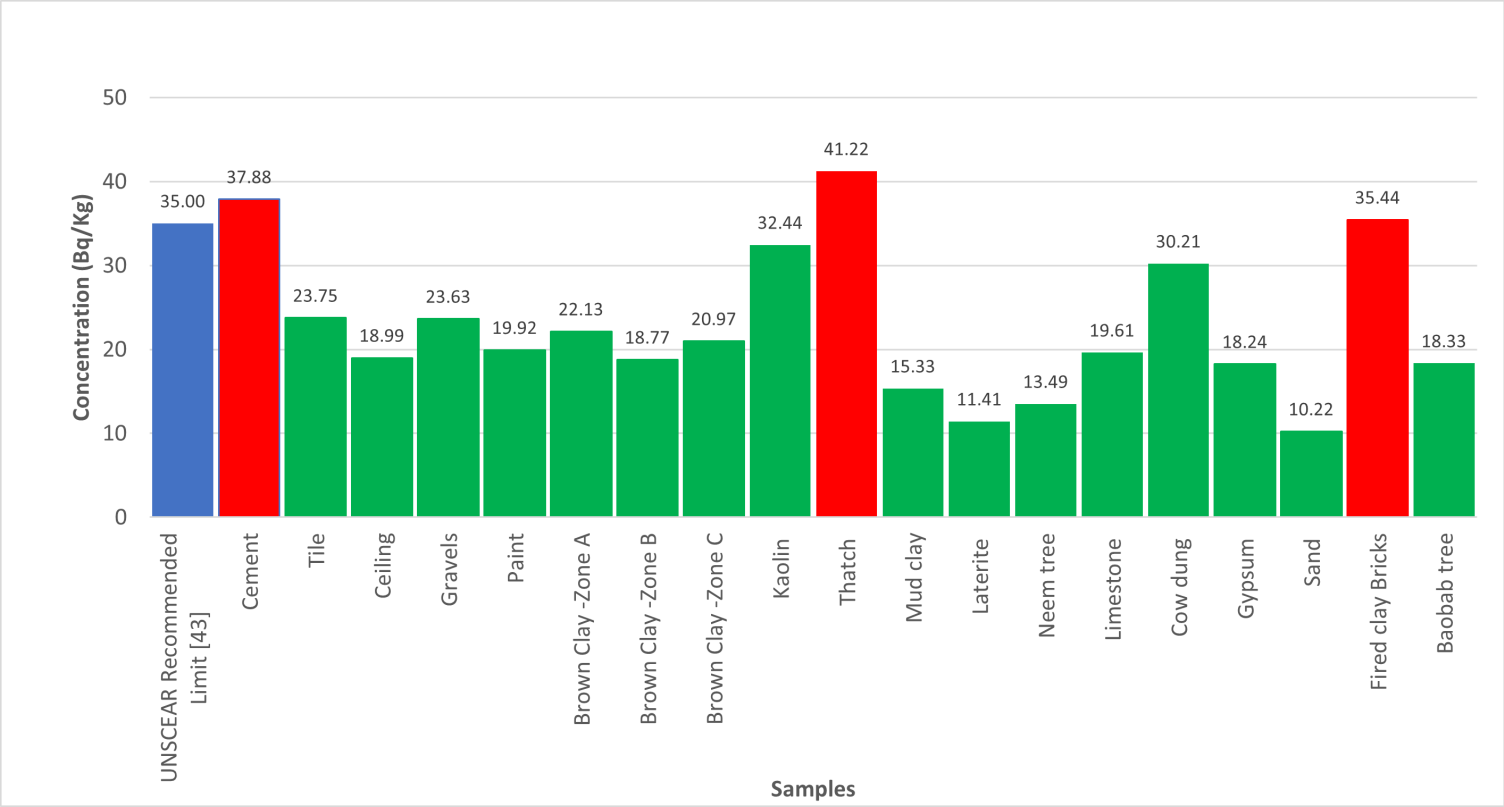
Illustrate the activity concentrations of $226_{\text{Ra}}$ across the analysed samples.

**Fig 3:**
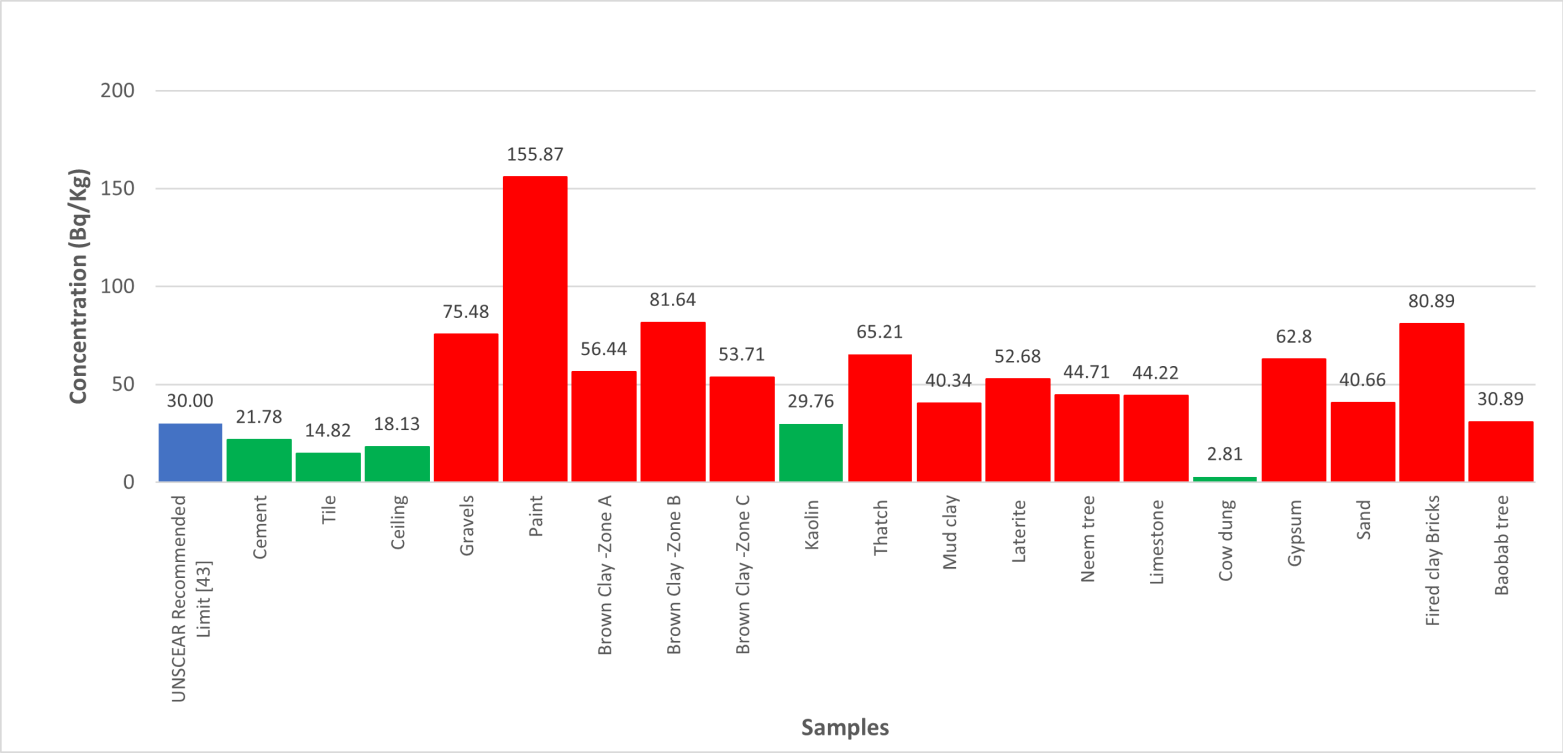
Illustrate the activity concentrations of $232_{\text{Th}}$ across the analysed samples.

As depicted in Fig 1, the significant $40_{\text{K}}$ activity concentration in Brown clay from Katsina Central and Gravels suggests that these materials may contribute to higher indoor radiation levels. The relatively high $226_{\text{Ra}}$ concentration in Cement, shown in Fig 2, also points to potential radiological hazards. The gamma radiation absorption rates further emphasize the need for careful selection of building materials to minimize indoor radiation exposure. The high $232_{\text{Th}}$ levels in Paint samples, illustrated in Fig 3, could pose long-term health risks if used extensively in indoor environments. Table 2 showed external hazard index found that the values range 0.1 to 0.7 with average value of 0.27 while the internal hazard index range 0.04 to 0.76 with a mean value of 0.22 and external hazard index with mean value 0.3107 which is less than average value of 1mSv/y recommended by UNSCEAR. The calculated value of the Gamma Index from Table 2, ranges from 0.22 to 0.53, and the average value of 0.53, this is lower than 1mSv/y (ICRP 12)

Table 3, in this table, Radium equivalent (Ra_eq_) obtained from the samples range between 86 to 263Bq/Kg with average value of 113.802Bq/Kg, absorbed dose and annual effective dose were recorded with mean values 51.171nGy/h and 0.06266mSv/y. These results are less than the safety limit approved by OECD.

**Table 3:** Radium equivalent, absorb dose and annual effective dose.

S/N	Sample	Radium equivalent (Ra_eq_) Bq/kg	Absorbed dose (D) nGy/h	Annual effective Dose Rate (E) mSv/y
1	Cement	86.8078	40.2735	0.04943
2	Tile	61.2725	28.6522	0.03515
3	Ceiling	58.0502	26.8999	0.03294
4	Gravel	168.6932	76.6146	0.09385
5	Paint	262.8809	115.0513	0.14087
6	Brown Clay -Zone A	140.5302	64.7437	0.07927
7	Brown Clay -Zone B	145.5862	63.4269	0.07767
8	Brown Clay -Zone C	125.5557	57.1569	0.06994
9	Kaolin	109.5679	51.9509	0.06371
10	Thatch	143.6011	63.2866	0.07745
11	Mud clay	95.3712	44.1614	0.05402
12	Laterite	104.0036	46.8484	0.05732
13	Neem tree	82.8141	36.1659	0.04423
14	Limestone	98.6896	44.1273	0.05398
15	Cow dung	41.7078	19.7092	0.02411
16	Gypsum	114.2978	49.7358	0.06083
17	Sand	73.6718	32.1681	0.03932
18	Fired clay Bricks	175.6608	78.2117	0.09598
19	Baobab tree	73.4939	33.0733	0.04062

**Fig 4:**
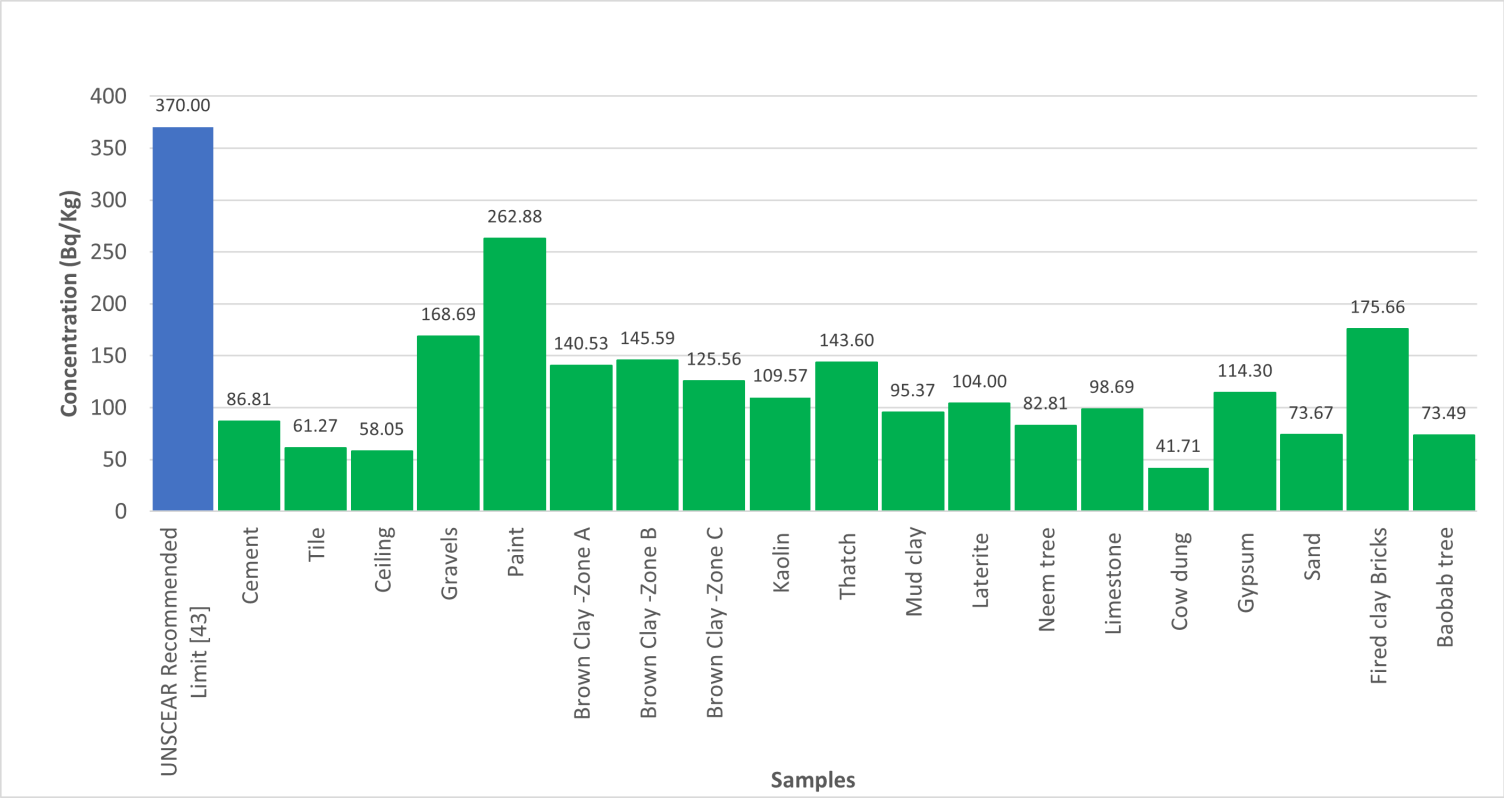
Shows the comparison of Radium equivalent with UNSCEAR Recommended limit.

**Fig 5:**
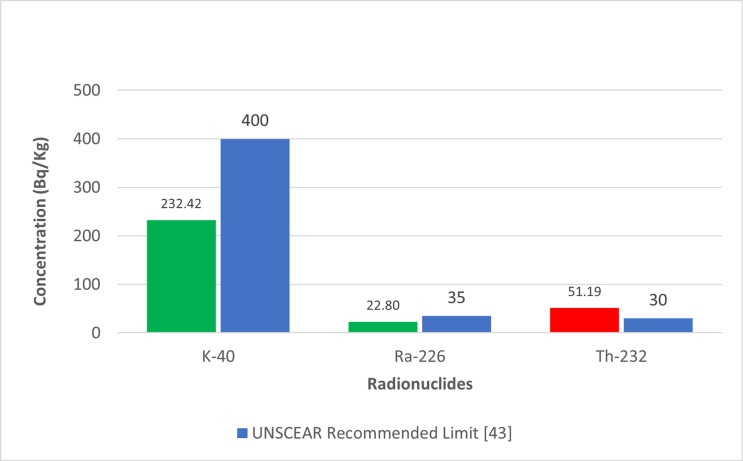
Shows the comparison of radionuclides activity concentrations with the recommended UNSCEAR Limit [ 41].

**Fig 6:**
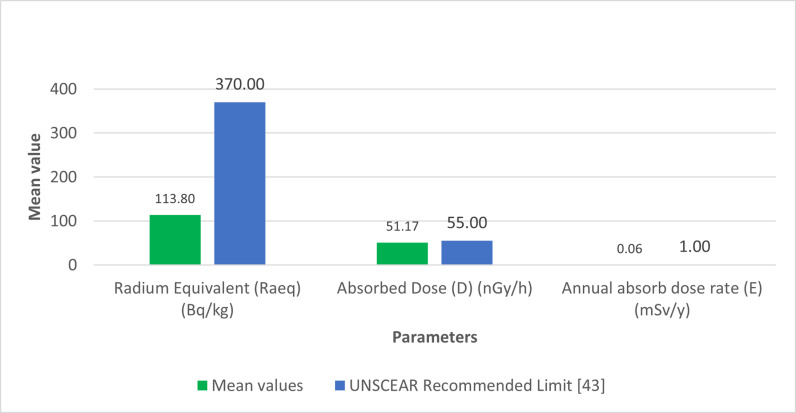
Shows the comparison of Radiological parameters with recommended UNSCEAR limit [ 41].

**Fig 7:**
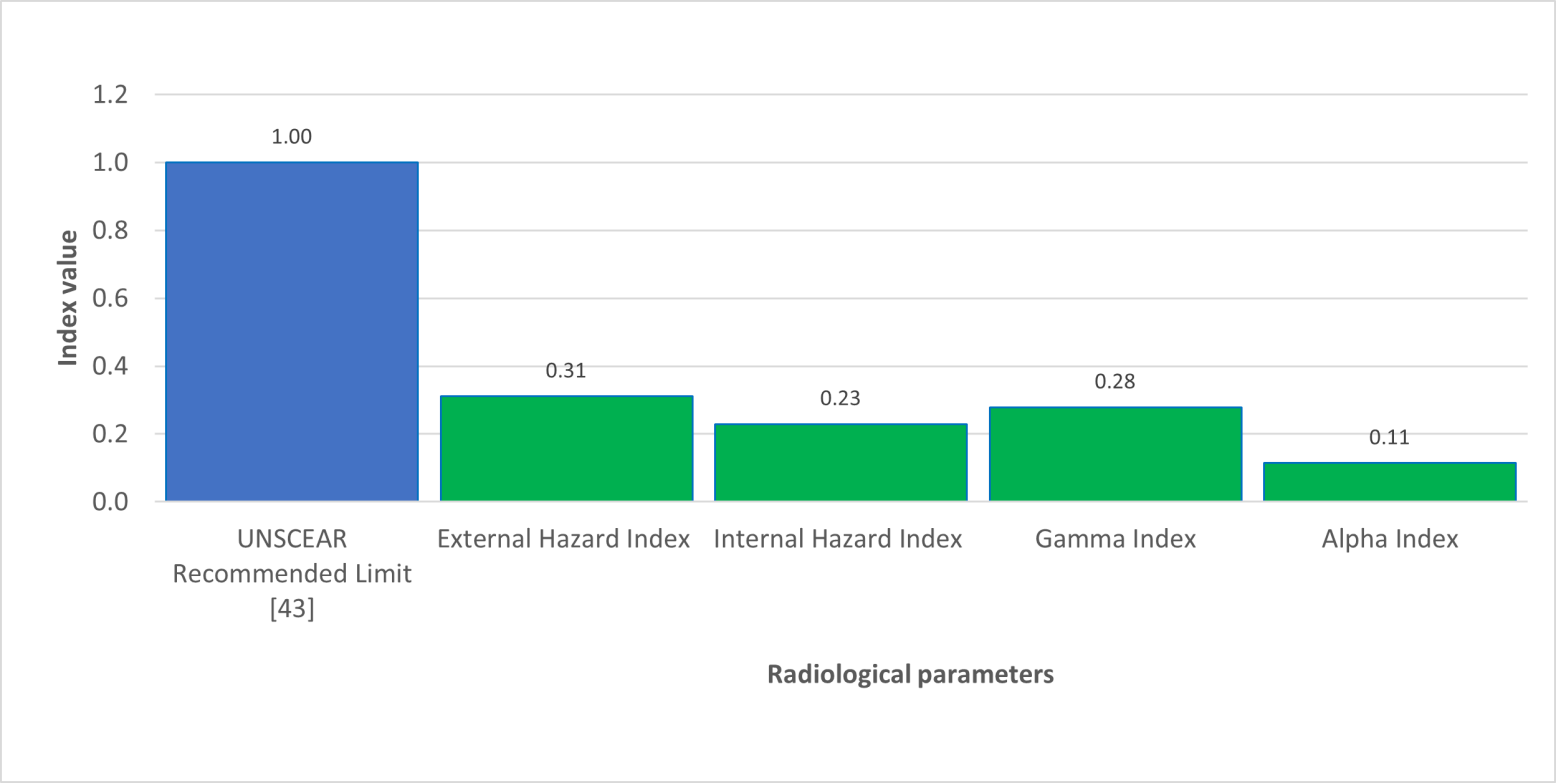
Comparison of gamma absorption with UNSCEAR Limit [ 41].

Fig 4 shows the comparison of Radium equivalent activity concentrations with UNSCEAR recommended limit [41]. Meanwhile, Fig 5 shows the comparison of radionuclides activity concentrations with the recommended UNSCEAR Limit [41]. In Fig 6, the comparison of radiological parameters with the recommended UNSCEAR Limit [41] are noticed. And, Fig 7 viewed the comparison of gamma absorption with UNSCEAR limit [41].

The statistical analysis was conducted using SPSS software version 27. The findings indicated that the mean activity levels of $40_{\text{K}}$, $226_{\text{Ra}}$ and $232_{\text{Th}}$ were 232.4211 Bq/kg, 22.7939 Bq/kg, and 51.1858 Bq/kg, with their corresponding standard deviation of 31.51075, 2.0584 and 7.75077 respectively. These values are presented in Table 4.

**Table 4:** Summary of Mean and standard deviation of Radionuclide Activity Concentrations.

Concentrations	Mean	Std. Error
$C_{40K}$ (Bq/Kg)	232.4211	31.5108
$C_{226Ra}$ (Bq/Kg)	22.7958	2.0584
$C_{232Th}$ (Bq/Kg)	51.1858	7.7508

The findings in Table 5 showed the gamma absorption from the samples were found to be 0.3107 and 0.2292, respectively. Additionally, the gamma index and alpha index were 0.2786 and 0.1140, respectively, indicating that these values are lower than the recommended limit of 1 mSv/y.

**Table 5:** Summary of the mean and standard deviation of Ra_eq_, absorb dose, and annual effective dose rates.

Parameters	Mean	Std. Error
Radium Equivalent (Ra_eq_) Bq/kg	113.8030	11.9069
Absorbed Dose (D) nGy/h	51.1715	5.1908
Annual absorb dose rate (E) mSv/y	0.0627	0.0064

The radium equivalent was recorded in Table 6, as 113.803Bq /kg. The absorbed and annual effective dose rate were 51.1 7 nGy/h and 0.0626mSv/y, respectively. These results were below the set limits of 500nGy/h and 1 mSv/y, respectively. However, Table 7 shows the comparison of activity concentration of radionuclides with another global studies.

**Table 6:** Summary of the mean and standard deviation.

Parameters	Mean	Std. Error
External Hazard Index	0.3107	0.0319
Internal Hazard Index	0.2292	0.0486
Gamma Index in mSGy/y	0.2786	0.0546
Alpha Index in mSGy/y	0.1140	0.0103
UNSCEAR Recommended Limit	1.0000	0.0000

**Table 7:** Comparison of activity concentration of radionuclides with another global studies.

S/N	Research	CK-40	CRa-226	CTh-232	References
1	Katsina State Northern Nigeria	232.421	22.796	51.186	Present study
2	Sokoto, Zamfara, and Kebbi State, Northern Nigeria	242.400	54.400	27.900	[34]
3	Ogun State (southern Nigeria)	620.89	68.03	84.79	[26]
4	Iran	75.78	8.69	3.30	[18]
5	Saudi Arabia	55.51	16.71	4.41	[1]
6	Red Sea, Saudi Arabia	403.31	-	9.38	[35]

## Conclusion

This study assessed the activity concentrations of naturally occurring radionuclides in building materials from Katsina State, Nigeria, and their potential health impacts. Using gamma-ray spectrometry, the mean activity concentrations were found to be 232.421 Bq/kg for potassium-40, 22.7959 Bq/kg for radium-226 and 51.1858 Bq/kg for thorium-232, all below UNSCEAR recommended limits [41]. The average radium equivalent activity $\left({\text{Ra}}_{\text{eq}}\right)$ was 113.80 Bq/kg, also below the safety threshold of 370 Bq/kg.

The gamma absorption rate was measured at 0.22, while the external and internal hazard indices were 0.3107 and 0.2292, respectively, both indicating low radiological risks. Notably, materials like Brown Clay and Gravels showed higher potassium-40., activity, while Cement exhibited elevated radium-226 levels, suggesting potential indoor radiation concerns.

Overall, the study concludes that the building materials analyzed do not pose significant health risks, but variability in radionuclide concentrations necessitates careful selection to minimize indoor radiation exposure.

In summary, the results from this study, indicate that the building materials analyzed in Katsina State pose no significant health hazard to the population. However, due to the variability in radionuclide concentrations, it is essential to carefully select building materials to minimize potential indoor radiation exposure.

### Recommendation

1. Monitoring and Regulation: Implement regular monitoring programs to assess radiation levels in building materials, especially those with elevated concentrations of radionuclides like $40_{\text{K }}$, $232_{\text{Th}}$, and $226_{\text{Ra}}$.
2. Public Awareness: Raise awareness among builders, homeowners, and the general public about the potential risks associated with natural radioactivity in building materials..
3. Use of Alternative Materials: Explore the use of alternative building materials that have lower concentrations of radionuclides.
4. Ventilation and Radon Mitigation: Enhance ventilation systems in buildings to reduce radon accumulation, which can significantly lower indoor radon concentrations.
5. Regulatory Framework: Develop or strengthen regulatory frameworks for construction materials to include limits on radionuclide concentrations.
6. Further Research: Encourage further research to expand knowledge on local geological conditions influencing radionuclide concentrations in building materials.
